# Sulphate of Cinchonia

**Published:** 1879-01-20

**Authors:** T. S. Lallerstedt

**Affiliations:** Ga.


### *SULPHATE OF CINCHONIA.*

BY T. S. LALLERSTEDT, M. D., OF GA.

I have been using cinchonia for nearly three years, and during the time I have not given one drachm of sulphate of quinine, but nearly two ounces of the sulphate of cinchonidia mostly for very young children. The reason I use the latter with children is that the cinchonia has a peculiar drying effect upon the tongue and other parts of the mouth and throat, and requires a little water frequently to moisten the parts.

In intermittent fever in all of its forms, in from four to six grain doses it breaks up the fever just as quick as the quinine, and I believe it is more permanent; in fact, I use it in all conditions wherein I formely used quinine There is one disadvantage attending the use of cinchonia; it is almost imposible to make it into pills and it will not form a solution, but I use it in combination with whatever would be compatible with the quinine. Cinchonia has a peculiar effect upon vision; after a person has taken from twenty-four to thirty grains he can see no one object long at a time; all things become as one; he cannot see to read; the pages of a book or paper become solid and dark.

I have no case book, but will give you a few cases from memory.

The first was an old lady, residing in Columbia county, Ga. She wrote me in March last she was down in bed with remittent fever, and no physician near. I sent her some cinchonia, and on receipt of it she took five-grain doses every three hours, until she had taken four doses, and in same way the next day. On the third day she was able to dispense with the medicine. She had been confined to her bed for eight days. Second, her neighbor had a son twenty years of age, suffering with chills and fever every day, and she sent them six powders with full directions how to use, and the chills were promptly cured, and there was no return.

On Sunday, Sept. 1st, 1878, I was called to see Mr. P., whom I found with very high fever, remittent type; pulse one hundred and forty, strong. He was taken on Friday, August, 30. I remained with him until the fever began to cool off, and I commenced giving sulphate of cinchonia grs. four, with dover's powders two grs. in combination, every two hours, with orders to continue unless the fever got very high. On Monday night, the 2d of September, being in his vicinity, I called to see him; he had a little fever since I left, but at that time was in a profuse perspiration. I left sufficient cinchonia for forty-eight hours. On morning 4th September, I called before day to see him; he was able to be up, and I dismissed him as cured.

I will relate a case of Mrs. Eliza G. I was called to see her on Sunday, 22d of September, '78, and found her suffering with a most agonizng pain in her right eye, very little fever. I decided she had what I term miasmatic neuralgia. I put her at once on sulphate of cinchonidia, grs. two and morphine sulphas grs. one-fourth, every half hour until she obtained relief. I then prepared twelve powders of cinchonidia grs. four, and opium pulv. grs. one-third each, to be given every three hours. I was called from home, and did not see her until Wednesday, 25th September, and found her very little better, I then substituted sulph. of cinchonia for cinchonidia and gave grs. v, every three hours, with same amount of opium. On Friday, 27th, found, her as she expressed it, feeling well.

In March, 1877, a lady got some boiling water poured into her shoe which made a very ugly place on the foot, and in the course of time it became very much inflamed, and very painful. She made an ointment of equal parts of cinchonia and dover's powders with glycerine, and applied it to the inflamed place, and in the course of two hours all the fever was gone. It was applied where the skin was not broken; it will not do where the skin is broken, for it gives too much pain. It was the only thing she could find that gave her any relief.

My success with the "Sulphate of Cinchonia," is just as good as when I used the sulphate of quinine. Cinchonia not being so soluble as quinine, will accomodate the stomach and bowels, and the impression will be felt much longer than the quinine upon the system, and you will therefore have to begin giving it sooner to ward off the attack. All of the unpleasant effects spoken of subside as soon as the system begins to relieve itself of the cinchonia, which is not very long.

The peculiar effects spoken of in the use of cinchonia are gathered both from experience upon myself, and from what others tell me.

